# Insight into Degrading Effects of Two Fungi on Polyurethane Coating Failure in a Simulated Atmospheric Environment

**DOI:** 10.3390/polym15020328

**Published:** 2023-01-09

**Authors:** Xiangping Hao, Kexin Yang, Dawei Zhang, Lin Lu

**Affiliations:** 1 National Materials Corrosion and Protection Data Center, Institute for Advanced Materials and Technology, University of Science and Technology Beijing, Beijing 100083, China; 2 BRI Southeast Asia Network for Corrosion and Protection (MOE), Shunde Innovation School, University of Science and Technology Beijing, Foshan 528399, China; 3 Beijing Advanced Innovation Center for Materials Genome Engineering, University of Science and Technology Beijing, Beijing 100083, China

**Keywords:** *T. funiculosus*, *P. chrysosporium*, polyurethane coating, penetration of mycelia, biodegradation

## Abstract

Two different fungi, *Talaromyces funiculosus* (*T. funiculosus*) and *Phanerochaete chrysosporium* (*P. chrysosporium*), were collected from the Xishuangbanna atmospheric corrosion site and incubated on a polyurethane (PU) coating at 30 °C for two weeks under 95% relative humidity (RH). The biodegrading effects of these fungi on the coating failure were investigated from aspects of metabolism and electrochemistry. The results showed that *T. funiculosus* contributed more to the degradation of the PU coating failure than *P. chrysosporium*, and two factors played dominant roles. First, the weight of the *T. funiculosus* mycelium was nearly 3 times more than that of *P. chrysosporium*, indicating there was more substrate mycelium of *T. funiculosus* deep into the coatings to get more nutrition in atmospheric during colonization. Second, *T. funiculosus* secreted carboxylic acids, such as citric, propanoic, succinic, and tartaric acids, and accelerated the hydrolysis of the ester and urethane bonds in the PU coatings. As a result, the mycelium of *T. funiculosus* readily penetrated the interface of the coating and substrate resulting in a rapid proliferation. Thus, the |Z|_0.01Hz_ value of the coating decreased to 5.1 × 10^4^ Ω·cm^2^ after 14 days of colonization by *T. funiculosus* while the value remained at 7.2 × 10^7^ Ω·cm^2^ after colonization by *P. chrysosporium*. These insights suggest that the biodegradation process in simulated atmospheric environments would provide theoretical guidance and directions for the design of antifungal PU coatings.

## 1. Introduction

Organic coating is the most efficient and portable method to inhibit the corrosion of metals by providing a physical barrier between the corrosive environment and the metal surface [1]. Polyurethane (PU) has been commonly used in anticorrosive organic coatings and is widely applied in a variety of atmospheric environments because of its excellent resistance to ozone aging, ultraviolet radiation, and atmosphere aging [2,3,4,5,6,7,8,9]. However, biodegradation of PU coatings by fungi is inevitable, as is degradation from atmospheric environments, especially in nutrient conditions such as tropical rainforests and marine environments [10,11,12,13].

Compared with the chemical degradation caused by environmental factors such as light and O_2_, degradation caused by fungi is a synergistic process of chemical and biological degradation. Recent studies have reported that fungi contribute significantly to the failure process of PU coatings by using PU as a carbon resource for its reproduction and degrading the coating through fungal metabolites [14,15,16]. Filip showed that PU acts as a sole nutrient source for the growth of microorganisms such as *Aspergillus niger* and *Cladosporium herbarum* [14]. Aguilar et al. showed that 22 fungal strains are capable of growing on PU by using it as a carbon resource, and all these strains have at least one enzymatic activity related to PU biodegradation with a common protease activity [15]. Besides enzymes secreted by the microbes, metabolic acids are one of the most important causes of damage to organic coatings. Sardon et al. summarized that organic acids such as formic acid, hydrochloric acid, sulfuric acid, etc., can cause amide bond scission and act as catalysts for the chemical degradation of polyamide [17,18,19]. Hepburn demonstrated that acidic conditions accelerate hydrolysis and the process is autocatalytic because carboxylic acid end groups are formed [20]. MacLeod et al. proposed that carboxylic acids have a stronger impact on plastic degradation than other organic acids [21]. Although hydrolysis of the ester bond of PU is the most prevalent degradation reaction [22], urea and urethane bonds can also be degraded by hydrolysis, but the degradation rate is slower [23]. The biodegrading effect of fungi on the polymer chains is explicated thoroughly in the literature, which can be summarized as the overall effect of fungi during the failure process of the coatings. This failure process of organic coatings includes four stages. Szociński et al. reported that the performance of protective organic coatings depends on its weakest area [24], which is the degradation area for ultraviolet radiation, heat, moisture, salt, and gas as well as atmospheric microorganisms [25]. Kothler demonstrated that water permeating through the organic coating from the weakest area establishes a conductive film of moisture and provides ions to carry the corrosion currents [26]. Water found under the cathodic blister is usually alkaline, which may cause stripping of coatings from the metal surface and generation of corrosion [27,28]. Hence, the failure process of organic coatings is not only correlated with the degradation at the surface but is a continuous process involving many interfaces.

Besides the penetrating effect of well-developed fungal mycelia, the atmospheric environment of a tropical rainforest plays an important role in biodegradation, as described in our previous work [29]. Negligence of this effect in previous studies can be attributed to the living environment of fungi. Most coatings are evaluated in liquid mediums, which shield a large amount of O_2_ and limit the free extension of mycelia, thereby affecting the fungal attack on the coating. Therefore, the results from liquid environments cannot be substituted for results obtained in atmospheric environments. Similarly, the antifungal coating based on liquids may not play a good role in atmospheric environment studies. In addition, because this environment is believed to be relatively mild, the coatings provide broad-spectrum protection rather than specific severe environments, evaluated with a uniform antifungal standard test (GJB150.10). Moreover, the strains used in the standard test are different from those present in severe environments, which may result in a different degrading effect [30,31,32]. Therefore, the antifungal properties of the coating in severe environments might be far from satisfactory [33]. Consequently, it is critical to evaluate the destructive effects of dominant fungi on coatings and the corresponding degrading mechanism in typical severe environments, which is a precondition for the development of highly efficient antifungal coatings.

In the present study, we employed two predominant fungi of the rainforest environment, *Talaromyces funiculosus* (*T. funiculosus*) and *Phanerochaete chrysosporium* (*P. chrysosporium*), which were collected from the atmospheric corrosion site in Xishuangbanna (southern China) and were different from previous work. The involvement of fungi in the electrochemical corrosion of the PU coating was investigated by scanning electron microscopy (SEM), high-performance liquid chromatography (HPLC), Fourier transform infrared spectroscopy (FTIR), and electrochemical impedance spectroscopy (EIS). Furthermore, along with the degradation of superficial coatings by organic acid secretions, the failure of the deep coatings caused by the mycelium penetration was also integrated in this research. The findings of this study would lay a solid foundation for developing high-efficiency antifungal coatings in an atmospheric environment.

## 2. Experimental Section

### 2.1. T. funiculosus and P. chrysosporium Spore Suspension

The fungi *T. funiculosus* and *P. chrysosporium* were isolated from an atmospheric test station in Xishuangbanna. The surface of the potato dextrose agar plate (PDA, Beijing Aoboxing Bio-tech. Co., Ltd., Beijing, China; potato 3 g/L, dextrose 20 g/L, agar 14 g/L) containing different mycelium was swiped with a sterile inoculation hook and put into 1 mL potato dextrose broth for incubation (PDB, Beijing Aoboxing Bio-tech. Co., Ltd., Beijing, China; potato 5 g/L, dextrose 15 g/L, peptone 10 g/L, NaCl 5 g/L; the broth was autoclaved at 121 °C for 15 min). The hyphae were filtered through four layers of sterile filter paper to obtain a spore suspension. Spore concentrations of *T. funiculosus* and *P. chrysosporium* were about ~10^6^ CFU/mL as determined by optical microscopy (AxioLab.A1, Zeiss, Oberkochen, Germany).

### 2.2. Growth Curve

The growth curves of both fungi were determined by the mycelium dry weight method. A spore suspension (3 mL) of *T. funiculosus* and *P. chrysosporium* was diluted with sterile deionized water and incubated for 2 weeks with shaking at 180 rpm/min. Fungal mycelia were collected after drying at 35 °C for 2 weeks.

### 2.3. Analysis of Organic Acid Metabolites

The dried mycelia were pulverized, extracted in ultra-pure water solution, and sonicated for 10 min. The suspension (1 mL) was filtered through a 0.45 µm pore size filter membrane and rinsed with 0.5 mL CH_3_OH. Detection of organic acid metabolites was performed by HPLC (LC-20AD, Shimadzu, Fukuoka, Japan) after pH maintenance. Chromatographic condition and the peak time of orgianc acids in the HPLC spectrum were exhited in Tables S1 and S2.

### 2.4. PU Coating Preparation

The Q235 steel coupons with dimensions of 60 mm × 40 mm × 1 mm were abraded with 240 grit silicon carbide paper, cleaned with acetone and ethanol in ultrasonic baths, and dried with N_2_. The PU coatings (Benchen, Shanghai, China) were prepared according to GB/T1765-89 and cured at room temperature, followed by sealing the other five sides with silicone sealant. The specimens were sterilized with UV light for 30 min before use.

### 2.5. Fungi Treatment Process

Spore suspensions (200 µL) of *T. funiculosus* and *P. chrysosporium* were spread separately on each specimen and incubated for 7 days in a constant temperature and humidity chamber (30 °C, 95% RH). An equal volume of sterile PDB was spread on the PU coatings as a control group. The morphologies of both fungi on the surface of the PU coatings were determined by SEM (JEOL, JCM6000-PLUS, Tokyo, Japan). The composition of the coatings immersed in the microbial culture medium for 7 days and 14 days was evaluated by FTIR (Thermo Scientific Nicolet iS5, Waltham, MA, USA).

### 2.6. EIS Test

EIS was carried out using an electrochemical station (PARSTAT 2273, Princeton, NJ, USA) with a three-electrode system in a 0.05% NaCl solution. The coated coupons after fungus treatment for different periods (30 °C; 95% RH) served as the working electrode. A platinum foil was used as the counter electrode, and a saturated calomel electrode (SCE) was used as the reference electrode. After stabilizing the system at open circuit potential (OCP), the EIS test of the coupons immersed in the medium was carried out in the frequency range from 10^5^ to 10^–2^ Hz along with a sinusoidal perturbation of 20 mV. All measurements were performed at least twice to ensure repeatability.

## 3. Results and Discussion

### 3.1. Morphologies and Growth Curve of T. funiculosus and P. chrysosporium

**Figure 1:**
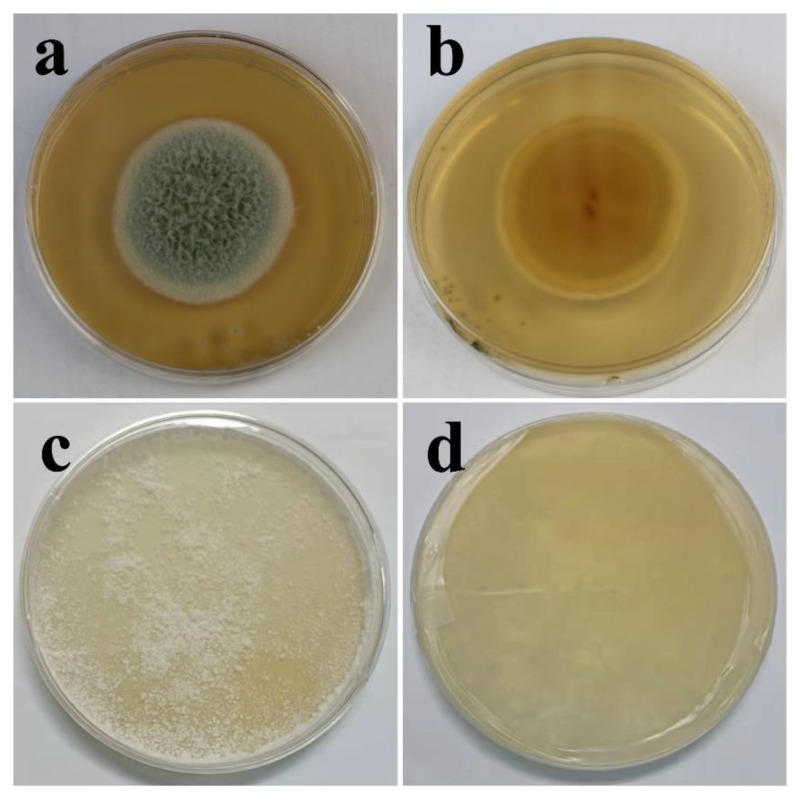
Characterization of *T. funiculosus* on PDA solid medium from the front view (**a**) and back view (**b**); characterization of *P. chrysosporium* on PDA solid medium from the front view (**c**) and back view (**d**).

The biological characteristics of fungi are associated closely with their living environments; thus, the liquid environment used to study the fungal effect on coatings shields a large amount of oxygen, causing the limited growth of mycelia (Figure S1, characterization of two fungi in PDB media). The morphologies of *T. funiculosus* and *P. chrysosporium* in PDA are shown in Figure 1. After 7 days of incubation, a round colony (45 mm diameter) of *T. funiculosus* on agar plates with a white edge was found, and the center of conidium was medium dark green from the front view, as shown in Figure 1a. The center of the colony (observed from the back of the plate) had reddish-brown dots with the color intensity decreasing while spreading outwards, as shown in Figure 1b. Moreover, the outermost circle of the colonies was the lightest, which could be the aerial mycelium. Compared with the characteristics of *T. funiculosus*, the colony features of *P. chrysosporium* on agar were different. As shown in Figure 1c, the white mycelium of *P. chrysosporium* was distributed throughout the entire agar instead of a circular colony. The deep layer of *P. chrysosporium* was also white when observed from the back of the plate without any other color pigmentation (Figure 1d).

**Figure 2:**
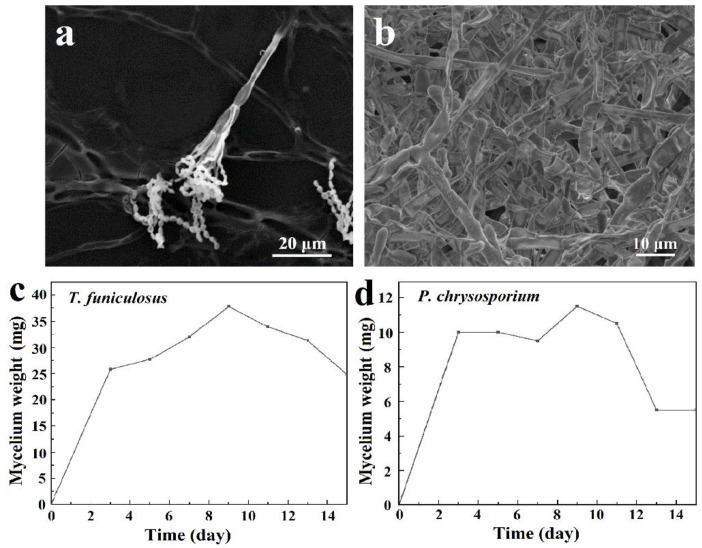
The SEM images of *T. funiculosus* (**a**) and *P. chrysosporium* (**b**); the growth curves of *T. funiculosus* (**c**) and *P. chrysosporium* (**d**) in the PDB culture medium.

The microstructures of these two fungi are shown in Figure 2a,b. The complete mycelia of *T. funiculosus* on the PU coating surface were about 50~100 µm as shown in Figure 2a. The mycelia spread on the coating surface had transverse septa, which divide the hyphae into several independent compartments. The sporulation structure was broom-like, scattering outwards, and the conidia were twin-whorled. The size of the oval-shaped conidium was about 1–5 µm, and dozens of conidia were clustered together. However, the mycelium of *P. chrysosporium* was multinucleated and had no septum or lock-like union, as shown in Figure 2b.

The growth curves of *T. funiculosus* and *P. chrysosporium* were determined by the mycelium dry weight method. As shown in Figure 2c,d, the curves could be divided into three stages: rapid growth stage, slow growth stage, and decline stage. From day 1 to day 3, the nutrients in the PDB medium were sufficient, and the spores absorbed those nutrients along with water in large quantities, resulting in a rapid increase in volume and mass. The mycelium weight increased to about 25.8 mg for *T. funiculosus* and about 10.0 mg for *P. chrysosporium*. As the nutrient content in the medium decreased, the space for the growth of fungi also decreased; hence, the growth rate slowed down. The mycelium weight of *T. funiculosus* increased from 25.8 mg on day 3 to 37.8 mg on day 9, while the mycelium weight of *P. chrysosporium* increased to 11.5 mg after incubation for 9 days. The mycelium weight of *T. funiculosus* was almost 3 times more than *P. chrysosporium*, implying that the growth and reproduction of *T. funiculosus* were better than *P. chrysosporium*. After 2 weeks of incubation, the mycelium weight of *T. funiculosus* and *P. chrysosporium* decreased to 24.8 mg and 5.5 mg, respectively, because of the harmful metabolites secreted by fungi such as propionic acid and less nutrition available in the substrate (mentioned in Section 3.2) [34]. This decrease could also be explained by autolysis of the dead cells, which is usually higher than the rate of cell regeneration. Theoretically, the growth phase of the mycelium also has a certain influence on the failure of the PU coatings, which will be discussed in Section 3.5.

### 3.2. Analysis of Organic Acids Metabolites

Table 1 and Table 2 show the types and contents of organic acids secreted by *T. funiculosus* and *P. chrysosporium* cultivated for 2 weeks. Content-1, Content-2, and Content-3 represent concentrations of the collected organic acids metabolites after 5 days, 9 days, and 2 weeks of incubation, respectively. Table 1 shows the collected organic acids after fungi cultivation for 5 days, which include citric acid, propanoic acid, succinic acid, tartaric acid, malonic acid, malic acid, acetic acid, lactic acid, methanoic acid, and oxalic acid. With increased incubation time, the total concentration of organic acids also increased from 331.36 µg/mL at 5 days to about 412.50 µg/mL at 2 weeks. Among these, citric acid, propanoic acid, succinic acid, and tartaric acid were present in the highest concentration, all accounting for about 81.78%, 86.11%, and 79.80% of the total organic acid concentration after incubation for 5 days, 9 days, and 2 weeks, respectively.

Table 2 shows the organic acids secreted by *P. chrysosporium*, which include oxalic acid, acetic acid, methanoic acid, propanoic acid, lactic acid, malonic acid, tartaric acid, malic acid, citric acid, and succinic acid. Compared with the increasing amount of total organic acids secreted by *T. funiculosus*, *P. chrysosporium* exhibited a slighter increasing trend with a total organic acid concentration of 323.84 µg/mL, 336.13 µg/mL, and 346.34 µg/mL after incubation for 5 days, 9 days and 2 weeks. The total concentration of organic acids secreted by *P. chrysosporium* after 2 weeks of incubation was less by 66.16 µg/mL than that of *T. funiculosus*. Moreover, the types of major organic acids secreted by *P. chrysosporium* were different from those of *T. funiculosus*. These included oxalic acid, acetic acid, methanoic acid, and propanoic acid, accounting for about 85.37%, 51.95%, and 59.73% of total organic acid content after incubation for 5 days, 9 days, and 2 weeks, respectively. Moreover, these four organic acids accounted for more than half of the total concentration of organic acids secreted by *P. chrysosporium*.

**Figure 3:**
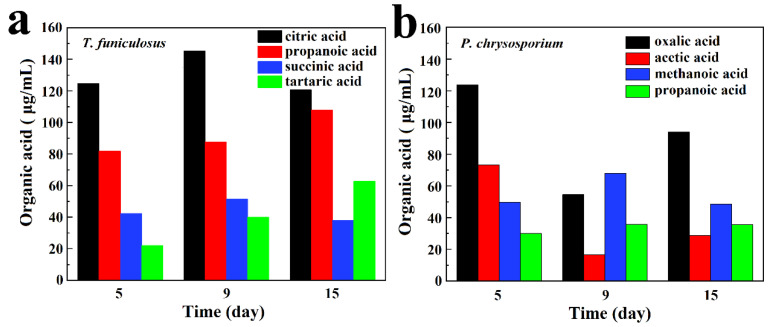
The main organic acid metabolites secreted by *T. funiculosus* (**a**) and *P. chrysosporium* (**b**) in different incubation periods.

The main four types of organic acids secreted by *T. funiculosus* and *P. chrysosporium* are demonstrated in Figure 3a,b. Figure 3a shows that organic acids with higher content were all carboxylic acids, including citric acid, propanoic acid, succinic acid, and tartaric acid. In addition, citric acid had the highest content in all incubation periods accounting for 46.03%, 44.79%, and 36.64% of the four major organic acids secreted by *T. funiculosus* at an incubation time of 5 days, 9 days, and 2 weeks, respectively. Propanoic acid content was the second highest followed by succinic acid content. The main organic acids secreted by *P. chrysosporium* were different, including oxalic acid, acetic acid, methanoic acid, and propanoic acid, and their contents kept changing constantly during the second week (Figure 3b). Noticeably, the content of oxalic acid increased again to about 93.92 µg/mL after incubation for 2 weeks. Oxalic acid is a toxic and corrosive substance for fungi that can impact the degradation of cells and acidify the cellular microenvironments [35], leading to the decreased reproduction of *P. chrysosporium* as shown in Figure 2d. As the total concentration of *P. chrysosporium* secretions and the amount of secreted oxalic acid decreased, the environment again became suitable for fungal reproduction and the mycelium weight increased after 7 days.

### 3.3. Morphological Analysis of Coating Surfaces

**Figure 4:**
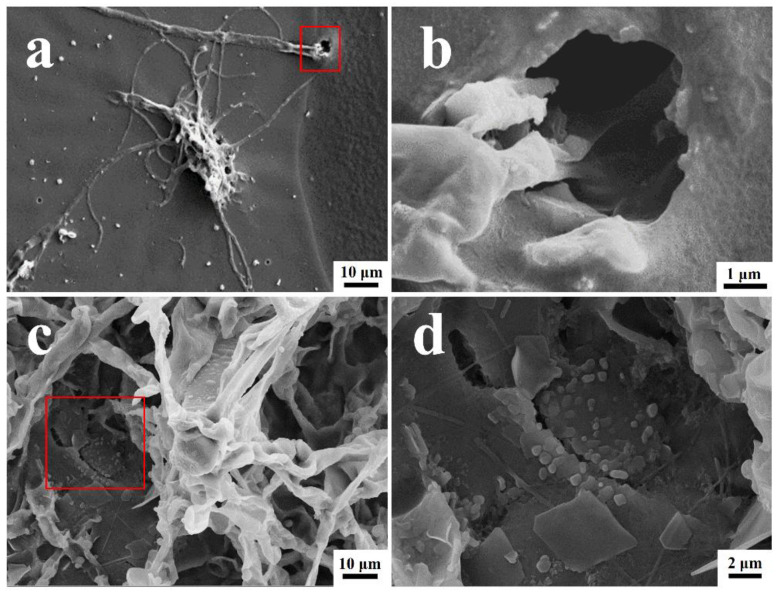
The SEM images of PU coatings surface morphology and their magnification after *T. funiculosus* (**a**,**b**) and *P. chrysosporium* (**c**,**d**) colonization for 7 days (35 °C, 95% RH).

The surface morphologies of the coated surfaces with and without the *T. funiculosus* and *P. chrysosporium* treatments are shown in Figure 4. The orginal state of the PU coting surface was as intact as shwon in Figure S3. As shown in Figure 4a, many *T. funiculosus* mycelia colonized on the coating surfaces and scattered a few spores nearby randomly after incubation for 7 days. Moreover, wrinkles and microholes were seen on PU coating near the mycelium and, as seen in the enlarged image (Figure 4b), mycelia extended down from a micro-hole in the coating surface to the metal substrate. This micro-hole may not only serve as a channel for transferring corrosive media but also provide conditions for the contact between the mycelium and metal substrates for establishing electron migration channels. Thus, it was established that the colonization of *T. funiculosus* on the coating surfaces could destroy the integrity of the coating and cause local damage, leading to a reduction in corrosion protection by the coating. While observing the morphology of the coating surface after treatment with *P. chrysosporium*, the fungi grew well, and the mycelium was reticulated over the PU coating surfaces (Figure 4c). The coating surfaces near the mycelium sank and developed cracks (Figure 4d). Such cracks were also observed on the surface of the PU coatings after treatment with *P. chrysosporium* mycelium, and scattered flakes were also seen near these cracks. The presence of such cracks and holes could cause a significant difference in the corrosion development on the metal surfaces after treatment with these two different fungi, which will be discussed later.

**Figure 5:**
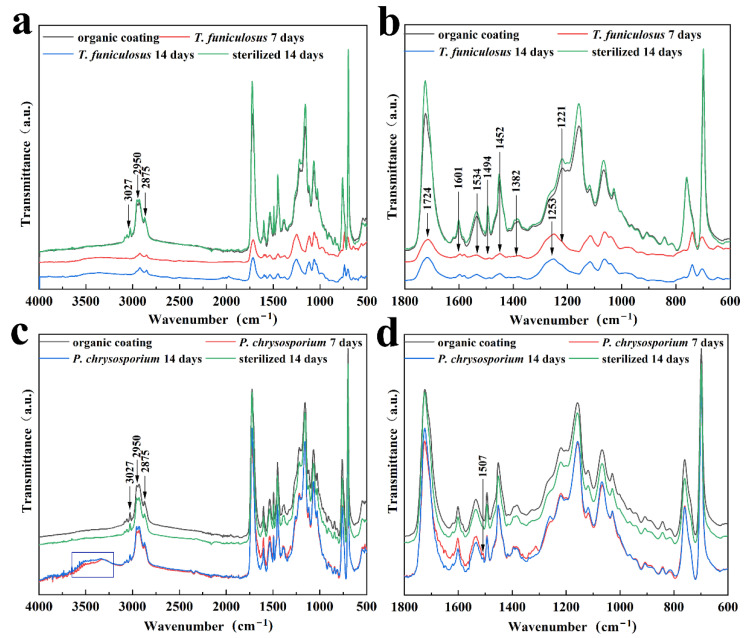
The FTIR spectra of PU coatings with and without treatment by sterilized and microbial PDB medium; with and without treatment by *T. funiculosus* (**a**,**b**), and with and without treatment by *P. chrysosporium* (**c**,**d**).

Further, FTIR was conducted to demonstrate the molecular structure changes in the PU coating after treatment with *T. funiculosus* and *P. chrysosporium* for 2 weeks. Initially, the impact of UV light on the PU coatings was evaluated by FTIR before fungi treatment (Figure S2). The results showed that the intensity and position of the characteristic peak of the PU coatings remained the same before and after the UV light sterilization process, leading to the conclusion that this process did not contribute to the degradation of such coatings. As seen in Figure 5a,b, the peaks at 1534 cm^–1^ and 1382 cm^–1^, which were assigned to the urethane bond (amide II and III), had weaker intensity than the sterilized counterpart, indicating that this bond was broken by *T. funiculosus*. It could be explained by the fact that microorganisms can utilize synthetic plastics as carbon and nitrogen sources [36]. Moreover, the secreted organic acids, especially carboxylic acid, may also contribute to urethane bond hydrolysis [17,37]. Moreover, the peaks at 2950 cm^–1^ and 2875 cm^–1^ (C–H) were also weaker in intensity than those of the original coating immersed in a sterile PDB medium, which means that the carbon chain in the PU coatings was destroyed by *T. funiculosus* [38]. Additionally, the intensities of the peaks at 1452 cm^–1^ and 1601 cm^–1^ almost disappeared, meaning that the benzene ring skeleton was also broken by *T. funiculosus.* The intensity of the peak at 1494 cm^–1^ decreased after incubation for 7 days and 2 weeks, respectively, but remained the same in the spectrum of the sterile sample after 2 weeks of incubation. According to previous reports, this peak is supposed to represent the energy provided for the fungi metabolism [36]. The corresponding absorption peak of the carbonyl and ester group also showed changes after treatment with *T. funiculosus*. It was observed that the intensity of the 1724 cm^–1^ peak (C=O, amide I) decreased, and the peak located at 1221 cm^–1^ almost disappeared in the FTIR spectra of *T. funiculosus* after 7 days and 2 weeks of incubation, respectively. Moreover, a new strong peak at 1253 cm^–1^ (C–O) appeared after colonization by *T. funiculosus*, revealing that carboxyl groups were formed after the ester bond hydrolysis. It can be explained by the speculation that the ester bond hydrolysis happens in the PU structure, and the carboxylic acids secreted by *T. funiculosus* can accelerate the hydrolysis of such bonds [22]. These results implied that along with the hydrolysis of ester and urethane bonds, carbon chains and ring structures were also broken by *T. funiculosus*.

The FTIR spectra of the PU coatings after incubation with *P. chrysosporium* for 7 days and 2 weeks were similar to the spectra of the control group and sterile group (Figure 5c,d). The intensities of the peaks located at 2950 cm^–1^ and 2875 cm^–1^ in these four spectra were almost equal, indicating that *P. chrysosporium* had no impact on the methyl or methylene groups. Moreover, the peaks located at 1724 cm^–1^ and 1221 cm^–1^ corresponding to the carbonyl and ester bonds showed almost no change compared to the control and sterilized counterparts. It means that the reason why the ester bond was not broken may be because the organic acids secreted by *P. chrysosporium* were not strong enough to destroy the ester bonds, or because the total amount of secreted acids was not enough to trigger the bond hydrolysis. In addition, the urethane bond was also not impacted by *P. chrysosporium* because the peaks at 1534 cm^–1^ and 1382 cm^–1^ had almost equal intensities in these four spectra (Figure 5d). Moreover, the hydrolysis of the urethane bond is more difficult than the ester bond [22,23]. Compared with the *T. funiculosus* treated sample, the PU coatings were able to maintain the bonds of the functional groups to keep their cross-linked structure. However, the presence of a broad peak around 3500~3300 cm^–1^ contributing to O–H indicated that the PU coating exhibited a water absorption phenomenon. Moreover, the new weak peak at 1507 cm^–1^ contributing to a nitro group showed that 3, 5-dinitrosalicylic acid was secreted by *P. chrysosporium* as a by-product [39].

### 3.4. EIS Analysis

**Figure 6:**
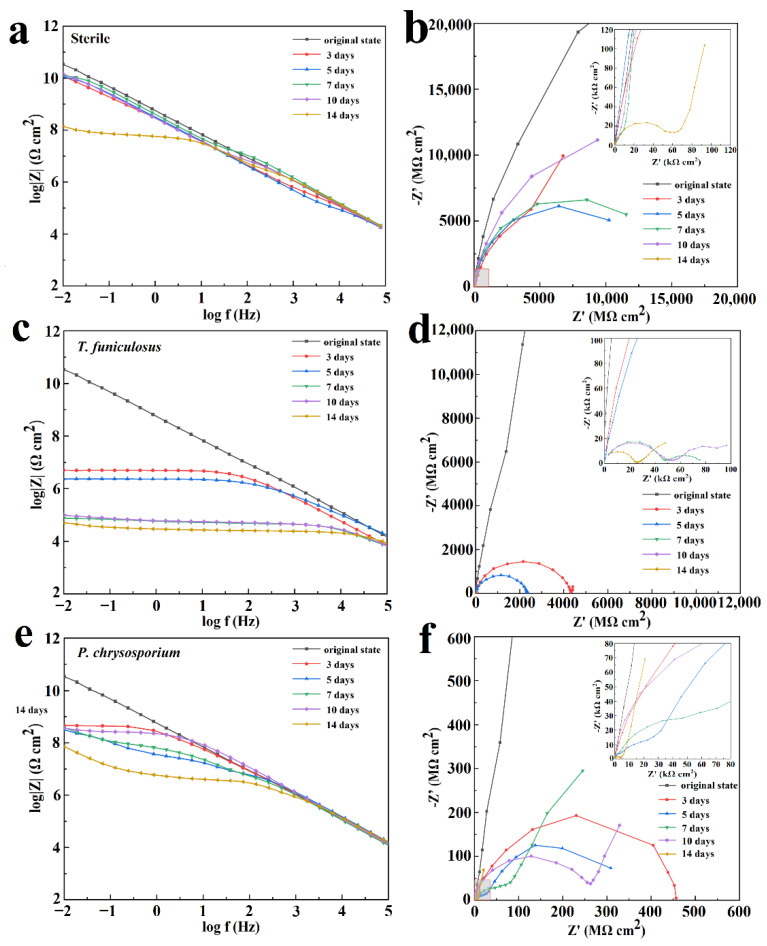
Bode plot and Nyquist plot of the PU coatings after treatment with sterile medium (**a**,**b**), with *T. funiculosus* (**c**,**d**), and with *P. chrysosporium* (**e**,**f**) in a humid and hot atmospheric environment for different periods.

EIS was utilized to evaluate the corrosion resistance of the PU coatings under the influence of *T. funiculosus* and *P. chrysosporium.* Figure 6 shows the Bode plot and Nyquist plot of EIS analyzed data for the PU coatings after 3 days, 5 days, 7 days, 10 days, and 14 days of fungi colonization in a humid and hot atmospheric environment. The impedance modulus at the low-frequency region (|Z|_0.01Hz_) is commonly used as a semi-quantitative indicator of the corrosion resistance properties of the coatings. The |Z|_0.01Hz_ value for the sterile condition was 3.6 × 10^10^ Ω·cm^2^ at an initial state (Figure 6a), indicating that the PU coating was intact with near-ideal barrier properties. Although the |Z|_0.01Hz_ value showed a slightly decreasing trend with the increasing time for the first 10 days, it was maintained over ~10^10^ Ω·cm^2^, which means that the PU coating had corrosion resistance properties in a humid and hot atmospheric environment. After 14 days, the |Z|_0.01Hz_ value decreased to 1.4 × 10^8^ Ω·cm^2^, indicating a decrease in the protective effects of the coatings but still maintaining a good anti-corrosion property. The size of the semi-circle in Figure 6b decreased slightly in the first 10 days, which is in agreement with the Bode plot results, revealing that the corrosive medium attempted to infiltrate into the PU matrix. The enlarged view of the high-frequency region in Figure 6b shows a capacitive reactance arc in the high-frequency region and a spread straight line in the low-frequency region of the Nyquist plot, indicating that the corrosive medium had penetrated the metal surface and the metal coupons had begun to corrode. However, because the PU coating still maintained its complete structure (Figure 5), the corrosive products would accumulate under the coatings.

After the colonization of *T. funiculosus* for 3 days and 5 days in the atmospheric environment, the |Z|_0.01Hz_ values decreased gradually to 5.1 × 10^6^ Ω·cm^2^ and 2.4 × 10^6^ Ω·cm^2^, respectively (Figure 6c). The size of the semi-circle also decreased in Figure 6d, indicating that the barrier properties of the coatings became weaker with increasing immersion time. After 7 days of treatment, the |Z|_0.01Hz_ values decreased dramatically to about 7.5 × 10^4^ Ω·cm^2^ and exhibited a slightly decreasing trend in the last 7 days reaching 5.1 × 10^4^ Ω·cm^2^ after 14 days of immersion. These results revealed that the coating lost its barrier properties, and corrosion occurred on the metal surface. The enlarged view of the high-frequency region in Figure 6d shows two capacitive reactance arcs and a gradual decrease in the arc radius with increasing immersion time. These results also demonstrated that the coatings lost their corrosion resistance properties after 7 days of colonization by *T. funiculosus* in the atmospheric environment.

In the *P. chrysosporium* treatment, the corrosion behavior of the coating was different compared to *T. funiculosus*. The |Z|_0.01Hz_ value decreased to 4.6 × 10^8^ Ω·cm^2^, indicating that the barrier performance of the coatings decreased but was still able to prevent the corrosive medium from infiltrating the metal surface (Figure 6e). After immersion for 5 days, the size of the arc in the low-frequency region was much larger than in the high-frequency region (Figure 6f), which represented corrosion inhibition properties and meant that the corrosion products could be accumulated on the surface of metal substrates. With the increase in immersion time, the accumulation of corrosion products led to a further decrease in |Z|_0.01Hz_ at 7 days and surpassed the counterpart values of 5 days. The size of the arc in the low-frequency region for 7 days of immersion was larger than that for 5 days of immersion (Figure 6f). These results implied that the generation of corrosion products could barricade the corrosive medium onto the metal surface resulting in a corrosion inhibition phenomenon at a low frequency. After 10 days of immersion, a Warburg impedance was created in the low-frequency region, indicating the formation of a diffusion layer made by the products at the interface. Meanwhile, the impedance modulus value in the Bode plot kept increasing in medium and low-frequency regions, implying that the corrosion products had barrier effects. The increase in the radius of the capacitive reactance arc at low frequencies verified this barrier phenomenon. After 14 days of fungi colonization, |Z|_0.01Hz_ decreased to 7.2 × 10^7^ Ω·cm^2^ (Figure 6f), which indicated that further deterioration of the coating happened and new corrosion occurred at the interface of coating and metal substrate.

### 3.5. Features of Coating Failure by Two Fungi

#### 3.5.1. Different Behaviors of Two Fungi

Combined with the results obtained in Section 3.1, Section 3.2, Section 3.3 and Section 3.4, the characteristic features of two fungi were disclosed and compared as follows:

(1) The mycelium stage of fungi and their quantity are determinants of their attack capability on the coating. The maximum mycelium weight of *T. funiculosus* and *P. chrysosporium* was about 37.8 mg and 11.5 mg, respectively, which means that the former possesses stronger abilities to obtain nutrients from coatings as well as to destroy the coating than the latter in atmospheric environments. Generally, the impact of fungi on the coating failure is proportional to their concentration, especially when PU is the only available carbon resource to support fungi metabolism in an atmospheric environment [36]. Moreover, better-developed mycelia could secret more organic acids, which was demonstrated by the higher total concentration of secreted organic acids by *T. funiculosus*.
(2) The types and total amount of organic acids secreted by *T. funiculosus* and *P. chrysosporium* were the key factors in the biodegradation of the PU coatings. The major four organic acids secreted by *T. funiculosus* were carboxylic acids, which can accelerate the hydrolysis of ester and urethane bonds. Comparatively, oxalic acid was the major organic acid secreted by *P. chrysosporium,* which was toxic to its metabolism, thus inhibiting or even decreasing the growth and reproduction of fungi during long incubation periods. In addition, ester and urethane bonds were barely affected, indicating that this fungus generated less damage to the chemical structure of the coating. Interestingly, the total amount of organic acids secreted by *T. funiculosus* increased constantly during the 2 weeks incubation time instead of remaining constant as did *P. chrysosporium*, which led to severe degradation of the PU coatings by *T. funiculosus*.
(3) The corrosion processes induced by these two fungi were different. According to the EIS results, the barrier properties of the coatings worsened after colonization by *T. funiculosus* than by *P. chrysosporium*, although the corrosion processes eventually occurred on the metal surface in both experimental groups. The cross-linked structures were destroyed by *T. funiculosus* colonization, decreasing the anti-corrosion strength of the coating. Further, the coatings were not able to resist the mycelium attack leading to perforations of the coating. Hence, the coatings’ failure not only occurs on the coatings’ surface caused by secreted organic acids but also in the deeper coatings caused by the mycelium attack. Moreover, the mycelium penetrating the coating can transport organic acids to the metal-coating interface and accelerate the process of corrosion. Hence, the EIS results showed that |Z|_0.01Hz_ decreased continuously, and the Warburg impedance was not present during the 14 days of treatment with *T. funiculosus*. However, after colonization by *P. chrysosporium*, the coating developed cracks and sank because atmospheric moisture permeated into the coatings and formed alkaline blisters [26], although the molecular structure inside the coating was intact. The |Z|_0.01Hz_ value remained stable from day 3 days to day 10 of colonization, and the Warburg impedance appeared, indicating that the corrosion products were accumulated under the coatings. During this process, although corrosion occurred at the metal-coating interface, the accumulated corrosion products inhibited the corrosion process of the metal substrate.

#### 3.5.2. Coating Failure Processes Associated with Two Fungi

**Figure 7:**
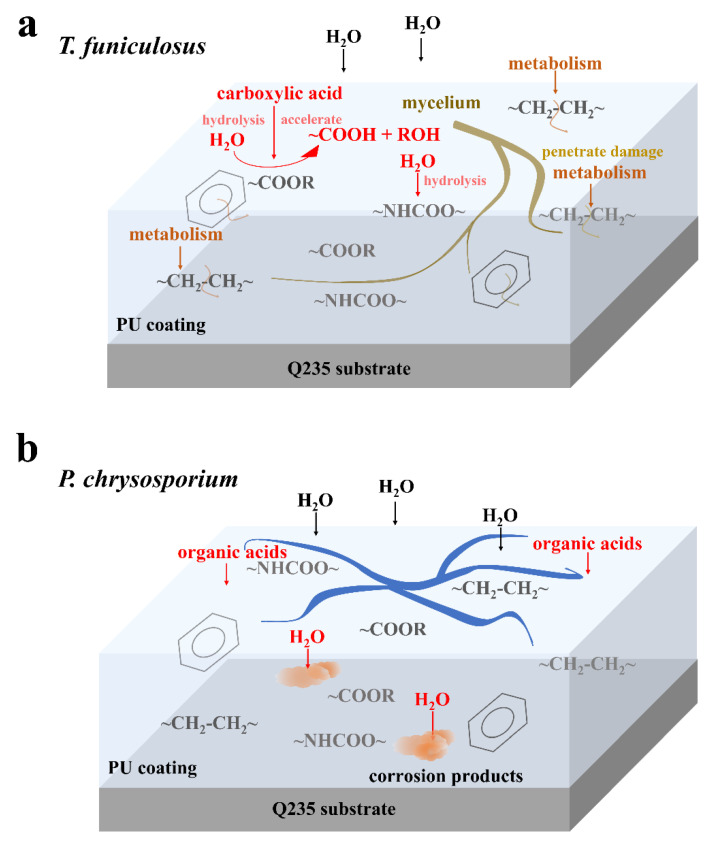
Diagram of the degradation mechanism in PU coatings by *T. funiculosus* (**a**) and *P. chrysosporium* (**b**) colonization.

The coating failure process due to *T. funiculosus* is shown in Figure 7a. Initially, the secretion of organic acids accelerates the hydrolysis of ester and urethane bonds, which decreases the strength of the coating and makes it less resistant to the physical attack of mycelium. As the fungus multiplies, the mycelium needs more nutrients and energy for metabolism and it extends deeper into the coatings, even penetrating through the coating to the metal surface. During this metabolic process, carbon chains and the C=C ring skeleton are damaged by *T. funiculosus*. Based on the penetration of mycelium, the ion migration channel could be established at the coating-metal interface [40], thereby accelerating the infiltration of the corrosive medium and deteriorating the metal surface even further. According to previous reports, an acidic environment and the secreted organic acids by fungi can dissolve iron impurities into iron ions and accelerate both anodic and cathodic polarization [41,42]. It means such channels allow the organic acids to reach the metal surface and may induce the dissolution of corrosion products, which was illustrated with a decreasing |Z|_0.01Hz_ during the ESI test. For the coating colonized by *P. chrysosporium,* no perforation was observed, but depressions and microcracks were seen (Figure 4). Although the corrosive medium permeates the coating matrix and corrodes the metal surface, the macromolecular organic acids could not reach the metal surface (Figure 7b) [20,21]. Moreover, the corrosion products can accumulate at the interface because of the absence of organic acids, thereby retarding the diffusion of electrolytes to some extent, as seen by the constant |Z|_0.01Hz_ value of ~10^8^ Ω·cm^2^ during this time.

## 4. Conclusions

In the present work, the biodegradation effects of two types of fungi (*T. funiculosus* and *P. chrysosporium*) on the corrosion failure of the PU coatings were investigated in a simulated atmospheric environment. The mechanism of the degradation process illustrated in this study is beneficial for proposing novel and precise solutions for developing and designing anti-corrosion PU coatings. The conclusion is summarized as follows.

(1) During the colonization of *T. funiculosus*, the mycelium penetrated the interface through microholes, while only depressions and cracks were observed in the *P. chrysosporium* colonized area.
(2) The total concentration of metabolic organic acid secreted by *T. funiculosus* and *P. chrysosporium* was about 412.50 µg/mL and 346.34 µg/mL, respectively. Citric acid, propanoic acid, succinic acid, and tartaric acid had the highest content in the metabolic organic acids secreted by *T. funiculosus,* while *P. chrysosporium* secreted oxalic acid, acetic acid, methanolic acid, and propanoic acid.
(3) The carboxylic acids secreted by *T. funiculosus* accelerated the hydrolysis of the ester and urethane bonds and damaged the carbon chain and benzene rings of the PU coating skeleton. Comparatively, acids secreted by *P. chrysosporium* had less impact on the degradation of the coating.
(4) The PU coatings almost lost the barrier properties after colonization by *T. funiculosus* for 14 days with the |Z|_0.01Hz_ value reaching about 5.1 × 10^4^ Ω·cm^2^. However, their corrosion inhibition properties were not affected by the colonization of *P. chrysosporium* for the same time and the |Z|_0.01Hz_ value observed was 7.2 × 10^7^ Ω·cm^2^.

## Figures and Tables

**Table 1:** The composition and contents of organic acids metabolites extracted from *T. funiculosus* after culturing for different days.

No.	Organic Acid	Content-1 (µg/mL)	Content-2 (µg/mL)	Content-3 (µg/mL)
1	citric acid	124.75	145.31	120.62
2	propanoic acid	81.85	87.55	107.96
3	succinic acid	42.36	51.50	37.89
4	tartaric acid	22.04	40.05	62.71
5	malonic acid	24.52	2.72	24.81
6	malic acid	13.69	14.51	23.68
7	acetic acid	10.99	11.85	11.62
8	lactic acid	0.87	11.89	11.34
9	methanoic acid	6.81	7.63	7.50
10	oxalic acid	3.49	3.74	4.38
Total		331.36	376.75	412.50

**Table 2:** The composition and contents of organic acid metabolites extracted from *P. chrysosporium* after culturing for different days.

No.	Organic Acid	Content-1 (µg/mL)	Content-2 (µg/mL)	Content-3 (µg/mL)
1	oxalic acid	123.65	54.50	93.92
2	acetic acid	73.17	16.45	28.77
3	methanoic acid	49.56	67.90	48.60
4	propanoic acid	30.09	35.78	35.57
5	lactic acid	19.43	45.31	28.86
6	malonic acid	22.62	20.90	2.17
7	tartaric acid	1.13	14.22	15.78
8	malic acid	0.00	4.05	38.41
9	citric acid	4.21	16.29	4.25
10	succinic acid	0.00	60.73	0.00
Total		323.84	336.13	346.34

## Data Availability

Data presented in this study are available on request from the corresponding author.

## Supplementary Materials

The following supporting information can be downloaded at: https://www.mdpi.com/article/10.3390/polym15020328/s1, Figure S1: Characterization of *T. funiculosus* (a) and *P. chrysosporium* (b) in PDB culture medium after 7 days; Figure S2: The FTIR spectra of intact PU coatings with and without treatment by UV light for 30 min. Wavenumber range of from spectrum 4000~500 cm^−1^ (a); from 1800~600 cm^−1^; Figure S3: The SEM image of intact PU coatings at the initial state; Table S1: Chromatographic condition; Table S2: The peak time of organic acids in the HPLC spectrum.
