# Supplementary material for: Insight into Degrading Effects of Two Fungi on Polyurethane Coating Failure in a Simulated Atmospheric Environment
Source: Polymers (Basel). 2023 Jan 9;15(2):328. doi: 10.3390/polym15020328 (PMC9866036; doi:10.3390/polym15020328)
Supplement: Supplementary file 1 [file polymers-15-00328-s001.zip › polymers-2108748-supplementary.pdf]

Supplementary Materials

In PBD culture mediums, the *T. funiculosus* are flocculent as shown in Figure S1a, while *P. chrysosporium* exists as small spheres with 0.5–1 cm diameter and is mainly concentrated at the bottom of the liquid environment (Figure S1b).

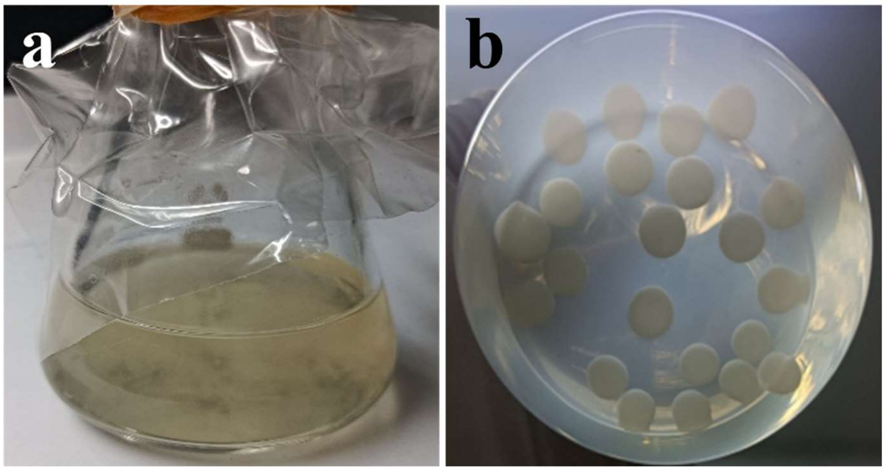

Figure S1. Characterization of *T. funiculosus* (a) and *P. chrysosporium* (b) in PDB culture medium after 7 days.

Table S1. Chromatographic condition.

Reagent	Content
chromatographic column	ultimate AQ-C18 4.6×250 mm 5 μm
mobile phase	20 mM Disodium hydrogen phosphate(pH 2.7) - methanol =99-1
flow velocity	0.7 mL/min
column temperature	30 °C
detection wavelength	210 nm
injection quantity	10 μL

Table S2. The peak time of organic acids in the HPLC spectrum**.**

No.	Organic Acid	Time (min)
1	oxalic acid	4.446
2	Tartaric acid	4.979
3	methanoic acid	5.405
4	Malic acid	6.227
5	malonic acid	6.433
6	lactic acid	7.450
7	acetic acid	7.993
8	citric acid	10.054
9	succinic acid	12.479
10	propanoic acid	18.181

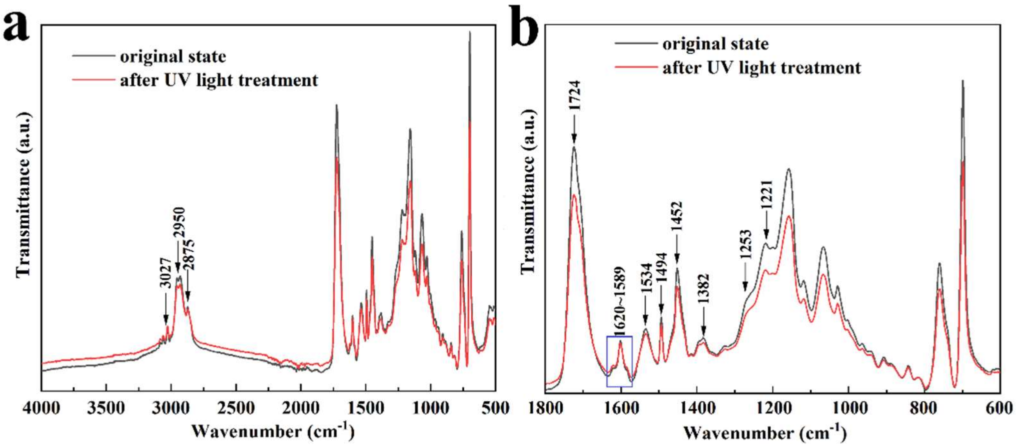

Figure S2. The FTIR spectra of intact PU coatings with and without treatment by UV light for 30 min. Wavenumber range of from spectrum 4000~500 cm^{-1} (a); from 1800~600 cm^{-1}.

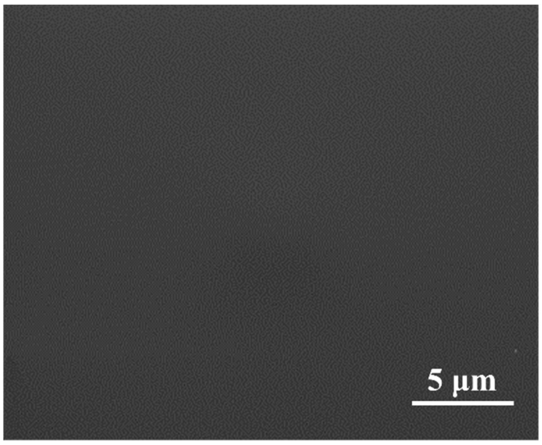

Figure S3. The SEM image of intact PU coatings at the initial state.
